# Supine, prone, right and left gravitational effects on human pulmonary circulation

**DOI:** 10.1186/s12968-019-0577-9

**Published:** 2019-11-11

**Authors:** Björn Wieslander, Joao Génio Ramos, Malin Ax, Johan Petersson, Martin Ugander

**Affiliations:** 1 Department of Clinical Physiology, Karolinska Institute, and Karolinska University Hospital, Stockholm, Sweden; 2 Department of Physiology and Pharmacology, Section of Anesthesiology and Intensive Care Medicine, Karolinska Institutet, and Function Perioperative Medicine and Intensive Care, Karolinska University Hospital, Stockholm, Sweden; 3 Kolling Institute, Royal North Shore Hospital, and Norther Clinical School, Sydney Medical School, University of Sydney, Sydney, Australia

**Keywords:** Cardiovascular magnetic resonance imaging, Pulmonary circulation, Posture

## Abstract

**Background:**

Body position can be optimized for pulmonary ventilation/perfusion matching during surgery and intensive care. However, positional effects upon distribution of pulmonary blood flow and vascular distensibility measured as the pulmonary blood volume variation have not been quantitatively characterized. In order to explore the potential clinical utility of body position as a modulator of pulmonary hemodynamics, we aimed to characterize gravitational effects upon distribution of pulmonary blood flow, pulmonary vascular distension, and pulmonary vascular distensibility.

**Methods:**

Healthy subjects (*n* = 10) underwent phase contrast cardiovascular magnetic resonance (CMR) pulmonary artery and vein flow measurements in the supine, prone, and right/left lateral decubitus positions. For each lung, blood volume variation was calculated by subtracting venous from arterial flow per time frame.

**Results:**

Body position did not change cardiac output (*p* = 0.84). There was no difference in blood flow between the superior and inferior pulmonary veins in the supine (*p* = 0.92) or prone body positions (*p* = 0.43). Compared to supine, pulmonary blood flow increased to the dependent lung in the lateral positions (16–33%, *p* = 0.002 for both). Venous but not arterial cross-sectional vessel area increased in both lungs when dependent compared to when non-dependent in the lateral positions (22–27%, *p* ≤ 0.01 for both). In contrast, compared to supine, distensibility increased in the non-dependent lung in the lateral positions (68–113%, p = 0.002 for both).

**Conclusions:**

CMR demonstrates that in the lateral position, there is a shift in blood flow distribution, and venous but not arterial blood volume, from the non-dependent to the dependent lung. The non-dependent lung has a sizable pulmonary vascular distensibility reserve, possibly related to left atrial pressure. These results support the physiological basis for positioning patients with unilateral pulmonary pathology with the “good lung down” in the context of intensive care. Future studies are warranted to evaluate the diagnostic potential of these physiological insights into pulmonary hemodynamics, particularly in the context of non-invasively characterizing pulmonary hypertension.

## Background

The influence of body position on the pulmonary circulation is incompletely understood [1], and can have important implications both for diagnosis and risk stratification in cardiopulmonary disease [2, 3], as well as therapeutic applications in surgery, anesthesia, and intensive care [1, 3–5]. Prior knowledge about right/left pulmonary blood flow distribution in the lateral position [6, 7] comes primarily from small studies in non-healthy anesthetized human subjects using intravenous injection of Xenon^133^ which may miss shunted blood flow [8–10], and animal studies performed on small sample sizes [1, 11]. Notably, these studies have yielded conflicting results [12, 13].

Non-invasive characterization of the pulmonary circulation by phase contrast (PC) cardiovascular magnetic resonance (CMR) has potential for improving screening, diagnosis, characterization, and risk stratification in pulmonary hypertension [13]. While right heart catheterization remains the reference standard, previous results suggest that mean pulmonary arterial pressure can be accurately estimated using 4D flow PC-CMR [14]. Also, CMR is ideally suited to quantify vessel cross-sectional area as a measure of pulmonary vascular distension.

2D PC-CMR has been used to measure the pulmonary blood volume variation (PBVV) during the cardiac cycle, as a surrogate of pulmonary vascular distensibility [15]. PBVV has been shown to be unchanged in systemic sclerosis patients who often have pulmonary arterial hypertension [16], but is reduced in left ventricular (LV) dysfunction in swine after experimental acute myocardial infarction [2]. This suggests that PBVV primarily reflects pulmonary venous or capillary distensibility, which may be affected in left-sided heart disease, whereas pulmonary arterial distensibility may be affected in pulmonary arterial hypertension. Consequently, PC-CMR may be able to non-invasively detect pulmonary hypertension and differentiate between pre- and post-capillary etiologies. Furthermore, the influence of body position upon PBVV is unknown. Altering body position can potentially serve as a “gravitational stress test”, which may unmask further information about the pulmonary circulatory hemodynamics.

In order to explore the potential clinical utility of body position as a modulator of pulmonary hemodynamics, we aimed to investigate the influence of body position on the distribution of pulmonary arterial and venous flow, pulmonary vascular distension, and pulmonary vascular distensibility using PC-CMR in healthy subjects.

Some of the results of these studies have been previously reported in the form of an abstract [17].

## Methods

### Study participants

This prospective study was conducted in non-smoking healthy subjects. Inclusion criteria were an age of 18–65 years, absence of prior cardiopulmonary or systemic disease, and absence of contraindications for CMR. Exclusion criteria were abnormal electrocardiography (ECG) findings, systolic blood pressure outside 90–140 mmHg, and pregnancy. Approval was obtained from the local ethics committee on human subject research, and all participants provided written informed consent.

### Screening

Immediately prior to the CMR scan, all patients underwent screening including standard 12-lead ECG (Marguette, General Electric Healthcare, Little Chalfont, United Kingdom), blood pressure measurement, and a patient history to exclude cardiopulmonary or systemic disease and contraindications to CMR.

### CMR acquisition

CMR was performed on a 1.5 T scanner (Aera, Siemens Healthineers, Erlangen, Germany) with ECG gating and phased array receiver coils, without any contrast agent. Participants sequentially underwent CMR scanning in four positions: supine, prone, and right, and left lateral decubitus. Flow measurements were obtained using a free-breathing velocity-encoded PC sequence with number of excitations set to three in order to reduce the impact of respirophasic variation. The total acquisition time was approximately 2.5 min depending on heart rate, representing approximately 30–40 respiratory cycles. Typical image acquisition parameters included 256 × 192–216 matrix size, 1.4 × 1.4 mm in-plane resolution, 5 mm slice thickness, TR/TE 30/2.7 ms. For all four body positions, flow was measured in the main pulmonary artery (velocity encoding 100 cm/s), right and left pulmonary arteries (velocity encoding 125 cm/s), and all four pulmonary veins (velocity encoding 80 cm/s). Phase contrast images were acquired during normal breathing and had a temporal resolution of 35 frames per cardiac cycle, roughly corresponding to 25–30 ms depending on heart rate. In order to image through-plane flow perpendicular to each vessel, a balanced steady state free precession (bSSFP) sequence was used to image the long axis of each vessel as previously described [15]. Upon acquisition, phase contrast images were screened for quality and image plane placement. Additional attempts were made until images were of acceptable quality and imaging plane. The full protocol for all sides including supine cine images and a two-minute break between body positions required approximately two hours. Typical scan duration for all pulmonary blood vessels in one body position was approximately 25 min. This included time for a localizer sequence, flow measurements in the main pulmonary artery, both pulmonary artery branches and all four pulmonary veins as well as, on average, one repeated attempt at imaging a pulmonary vein that was difficult to visualize. We estimate that unilateral measurements including one artery and two pulmonary veins can be obtained in ~ 8–10 min for one side in a clinical setting.

Furthermore, the imaging protocol performed in the supine position included 2-, 3-, 4-chamber long axis bSSFP cine images of the LV as well as a short-axis stack. Typical image parameters for bSSFP images included 256 × 216 matrix size with 1.5 × 1.5 mm in-plane resolution, TR/TE 36/1.16 ms.

### Image analysis

**Fig. 1:**
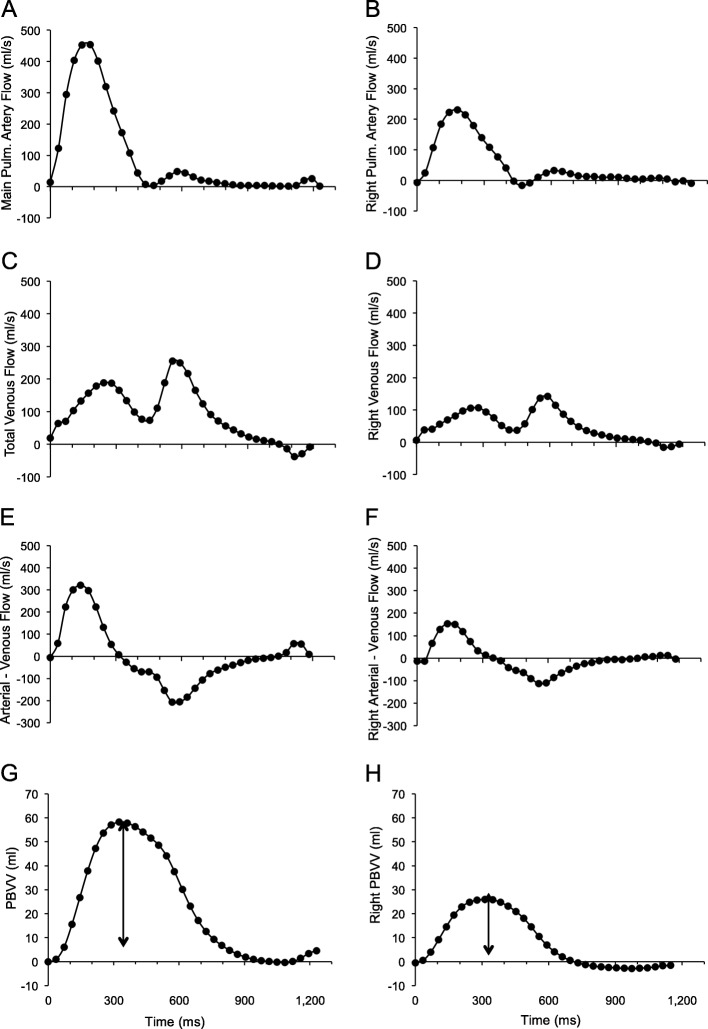
Panels A-H illustrate the method for using cardiovascular magnetic resonance (CMR) flow measurements to measure pulmonary blood volume variation (PBVV) for both lungs combined (**a**, **c**, **e**, **g**), and separately as represented by the right lung (**b**, **d**, **f**, **h**), in a representative subject. All plots show a given measure in relation to time during the cardiac cycle. Panels A and B show blood flow in the main pulmonary artery and right pulmonary artery, respectively. Panels **c** and **d** show total pulmonary venous flow and right pulmonary venous flow, respectively. Panels **e** and **f** show difference between arterial and venous flow for each time frame, indicating the net inflow and outflow, respectively, to or from the pulmonary circulation for each time frame. Panel **g** and show the cumulative integral of the difference in blood flow from panels **e** and **f** over time, corresponding to the change in pulmonary blood volume in both lungs and the right lung, respectively, over one cardiac cycle. The vertical arrow shows the PBVV in ml

Image analysis was performed using the freely available software Segment (version 2.0 R5039, Medviso, Lund, Sweden) [18, 19]. LV volumes, mass, and ejection fraction were measured from the cine short-axis stack obtained in the supine position using manual delineation (Table 1). Compensation for eddy currents was applied using a quadratic fit to stationary background tissue. Vessels were manually delineated and the region of interest was automatically propagated throughout the cardiac cycle. The accuracy of the vessel delineation in all time frames was manually verified. Flow was measured in milliliters per second (ml/s) for each time frame, which was then used to calculate net flow per cardiac cycle per vessel, and PBVV, as previously described [2, 15]. The methodology for the calculation of PBVV is illustrated in Fig. 1. In brief, the net inflow and outflow of blood to and from both lungs was calculated per time frame by subtracting the summed venous flow from the pulmonary arterial flow, and multiplied by time frame duration. The net in/outflow of consecutive time frames was calculated and plotted to yield the cumulative pulmonary blood volume change over time. The PBVV was calculated as the difference in ml between the maximum accumulated pulmonary net inflow and outflow, respectively. Relative PBVV was calculated by dividing the PBVV by the net pulmonary arterial flow volume over the cardiac cycle. Both PBVV and relative PBVV were calculated for both lungs independently, and together, in all four body positions. Cardiac output was calculated as the product of heart rate and stroke volume from flow measurement in the main pulmonary artery. Cross-sectional vessel areas were measured in PC-CMR images in all time frames, and averaged over the cardiac cycle. Localizer image stacks were visually examined for regional pulmonary areas with increased signal intensity, as a sign of atelectasis. As an internal control of data consistency, we compared the stroke volume derived from cine short axis stacks of the LV to the measured flow in the main pulmonary artery.

**Table 1:** Subject characteristics

Number, *n*	10
Female gender, *n* (%)	5 (50)
Age, years	27 ± 1
Height, cm	177 ± 12
Weight, kg	72 ± 12
BMI, kg/m^2^	23.0 ± 0.5
BSA, m^2^	1.9 ± 0.1
QRS duration, ms	96 ± 3
Heart rate, beats/min	61 ± 3
CMR parameters	
Cardiac output, l/min	6.2 ± 0.3
Cardiac index, l/min/m^2^	3.3 ± 0.2
LVEF, %	60 ± 2
LVM, g	105 ± 7
LVMI, g/m^2^	55 ± 2
LVESV, ml	76 ± 8
LVESVI, ml/m^2^	39 ± 3
LVEDV, ml	187 ± 12
LVEDVI, ml/m^2^	98 ± 4

Continuous variables are presented as mean ± SEM. Proportions are presented as percentages

Abbreviations: *BMI* Body mass index, *BSA* Body surface area, *CMR* cardiovascular magnetic resonance, *ECG* electrocardiogram, *LVEDV* Left ventricular end-diastolic volume, *LVEDVI* Left ventricular end-diastolic volume divided by BSA, *LVEF* Left ventricular ejection fraction, *LVM* Left ventricular mass, *LVMI* Left ventricular mass divided by BSA

### Terminology

Terms and their definitions as used in this study are listed in Table 2.

**Table 2:** Terminology

Term	Definition
Dependent	When a subject is lying in the lateral position, the term *gravity-dependent* or simply *dependent* lung refers to the lowermost lung, which is also referred to as being in the *dependent* position.
Non-dependent	When a subject is lying in the lateral position, the term *gravity-non-dependent* or simply *non-dependent* lung refers to the uppermost lung, which is also referred to as being in the *non-dependent* position.
Distension	Generally refers to an enlargement, dilation, or ballooning effect. In this context we use *distension* to refer to an increase in intra-vessel blood volume. We used the changes in vascular cross-sectional area as a measure of changes in vessel *distension*.
Distensibility	Generally refers to the capacity to distend as a result of pressure from inside. In cardiovascular physiology, vascular *distensibility*, or compliance, refers to the change in intravascular volume per unit of change in intravascular pressure. In the current study, we used PBVV as a marker of *distensibility*.
Pulmonary blood volume variation (PBVV)	The cumulative change in intravascular blood volume in the pulmonary circulation that occurs during the cardiac cycle. This is measured in ml, by using CMR flow imaging to measure the net inflow and net outflow of blood into and out of the pulmonary circulation. *PBVV* can be measured either combined for both lungs, or independently for each respective lung.
Relative PBVV	The *PBVV* in ml, divided by the arterial flow volume per heartbeat in ml. Thus*, relative PBVV* is a fraction which in this study is expressed as a percentage.
Pulmonary vascular distensibility reserve (PVDR)	The pulmonary vascular distensibility reserve (*PVDR*) was defined as the percent change in unilateral *relative PBVV* when changing between body positions. If supine left lung *PBVV* = 25 ml and left pulmonary artery net flow = 50 ml, then left *relative PBVV* = 50%. If in the same subject, in the right lateral position, left lung *PBVV* = 32 ml and left pulmonary artery net flow = 40 ml, then left *relative PBVV* = 80%. Left PVDR is then calculated as: *∆ relative PBVV* / supine *relative PBVV* = (80–50)/50 = 60%.

### Statistical analysis

Statistical testing was performed using SPSS Statistics (version 24, Statistical Package for the Social Sciences (SSPS), International Business Machines, Inc., Armonk, New York, USA). Continuous variables were reported as mean ± standard error of the mean (SEM). Categorical variables were presented as percentages. Differences in continuous variables were tested using the Wilcoxon signed-rank test or the Mann-Whitney U test, as appropriate. Differences in repeated measures between multiple groups was tested with the Friedman test. A *p*-value less than 0.05 was considered statistically significant. Due to the uncertainty of effect magnitudes and measurement variation in the impact of body position of PBVV and relative PBVV, no a priori power calculations were made. We measured the interobserver variability for PBVV, relative PBVV for the left lung in the supine and the right lateral positions, as well as for left lung PVDR, by having a second observer perform independent image analysis on the same raw data. The interobserver variability is reported as the mean percent difference and standard deviation from the average of the two observers, as the Dahlberg error, relative Dahlberg error and as the intra-class correlation coefficient (ICC).

## Results

### Study participants

The study participants were 27 ± 1 years (range: 22–31 years), and were equally distributed between the sexes. Subject characteristics are summarized in Table 1. There were no signs of atelectasis in any subject in any body position. We found no pulmonary vein abnormalities or unusual variations in our cohort, including the presence of any separate right middle lobe vein.

### Impact of body position on cardiac output

**Fig. 2:**
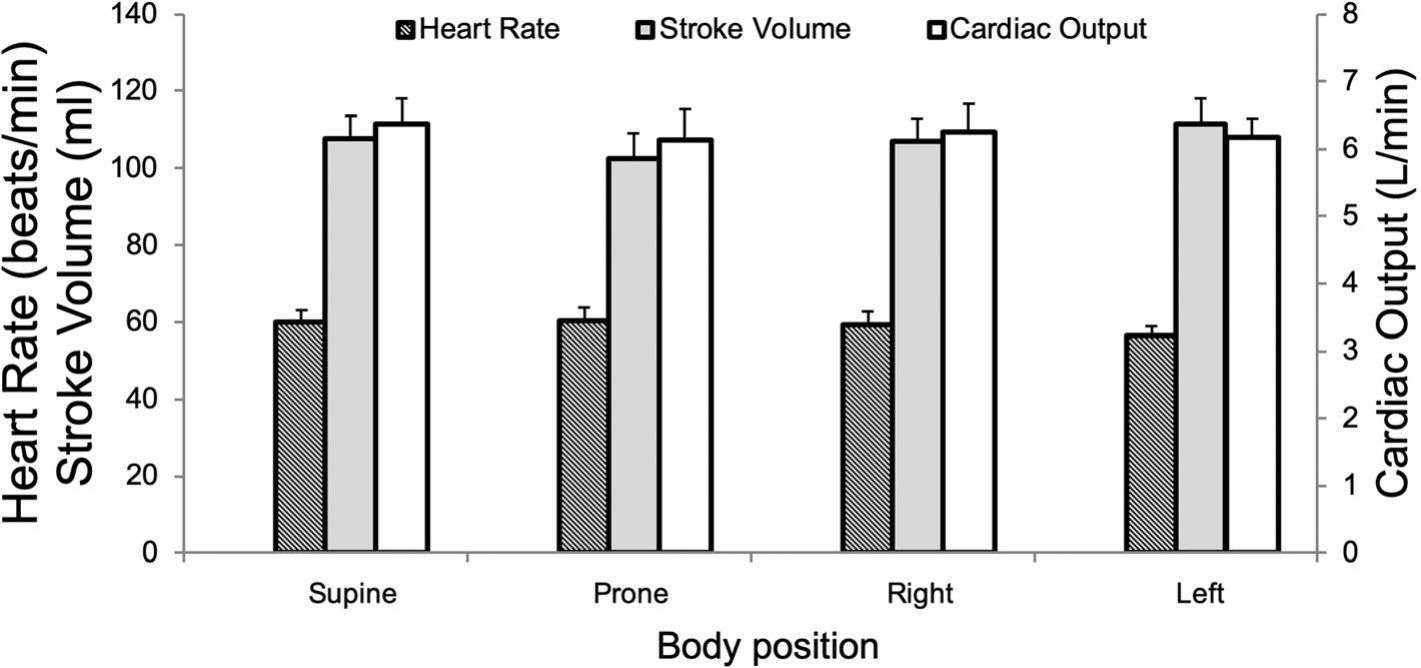
Neither heart rate, stroke volume as measured in the main pulmonary artery nor cardiac output differed across different body positions, Frieder’s test *p* > 0.05 for all three. Error bars indicate standard error of the mean (SEM)

Neither stroke volume (*p* = 0.12), heart rate (*p* = 0.09), cardiac output (*p* = 0.84) nor cardiac index (p = 0.84) differed across body positions, as shown in Fig. 2. There was no difference between flow volume in the main pulmonary artery and the summed flow in the pulmonary veins in the supine position (*p* = 0.85), indicating the absence of systematic flow errors or bronchopulmonary shunts in the group as a whole. We found no difference between cine-derived LV stroke volume (111 ± 6 ml) and net flow volume measured in the main pulmonary artery (106 ± 6, *p* = 0.57).

### Impact of body position on pulmonary blood flow distribution

**Fig. 3:**
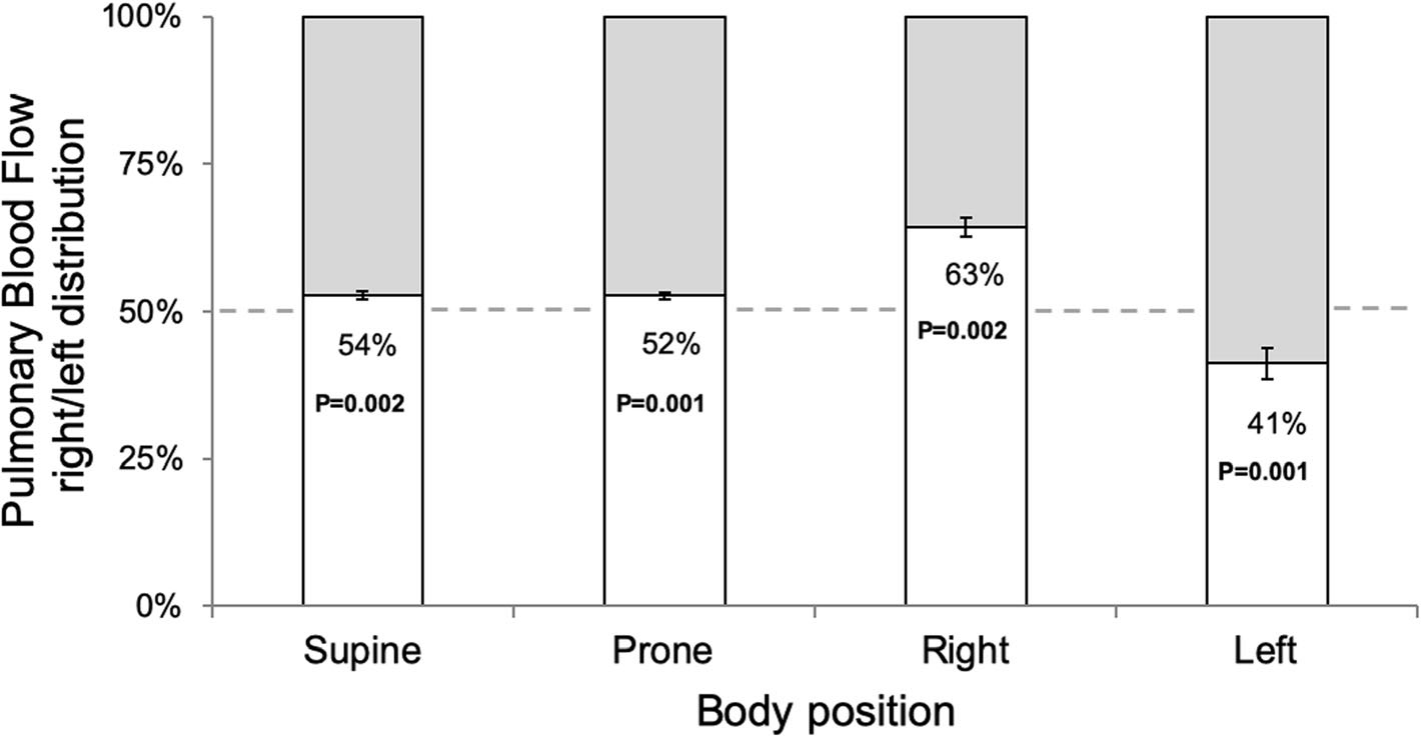
Proportion of pulmonary blood flow volume going to the right lung, shown in each tested body position. The white bars and percent numbers represent the percent distribution of flow to the right lung. The gray region represents the percent distribution to the left lung. Error bars represent standard error of the mean (SEM). *P*-values reflect Wilcoxon signed-rank test with even distribution as the null hypothesis

**Fig. 4:**
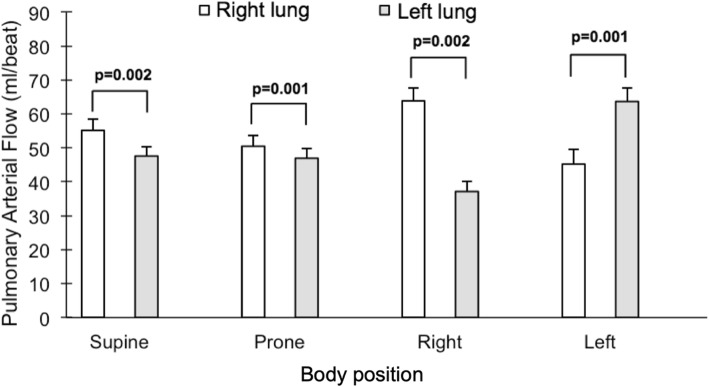
Comparison between the pulmonary blood flow volumes in each body position, for the right lung (white bars) and the left lung (gray bars). Error bars indicate standard error of the mean (SEM)

**Fig. 5:**
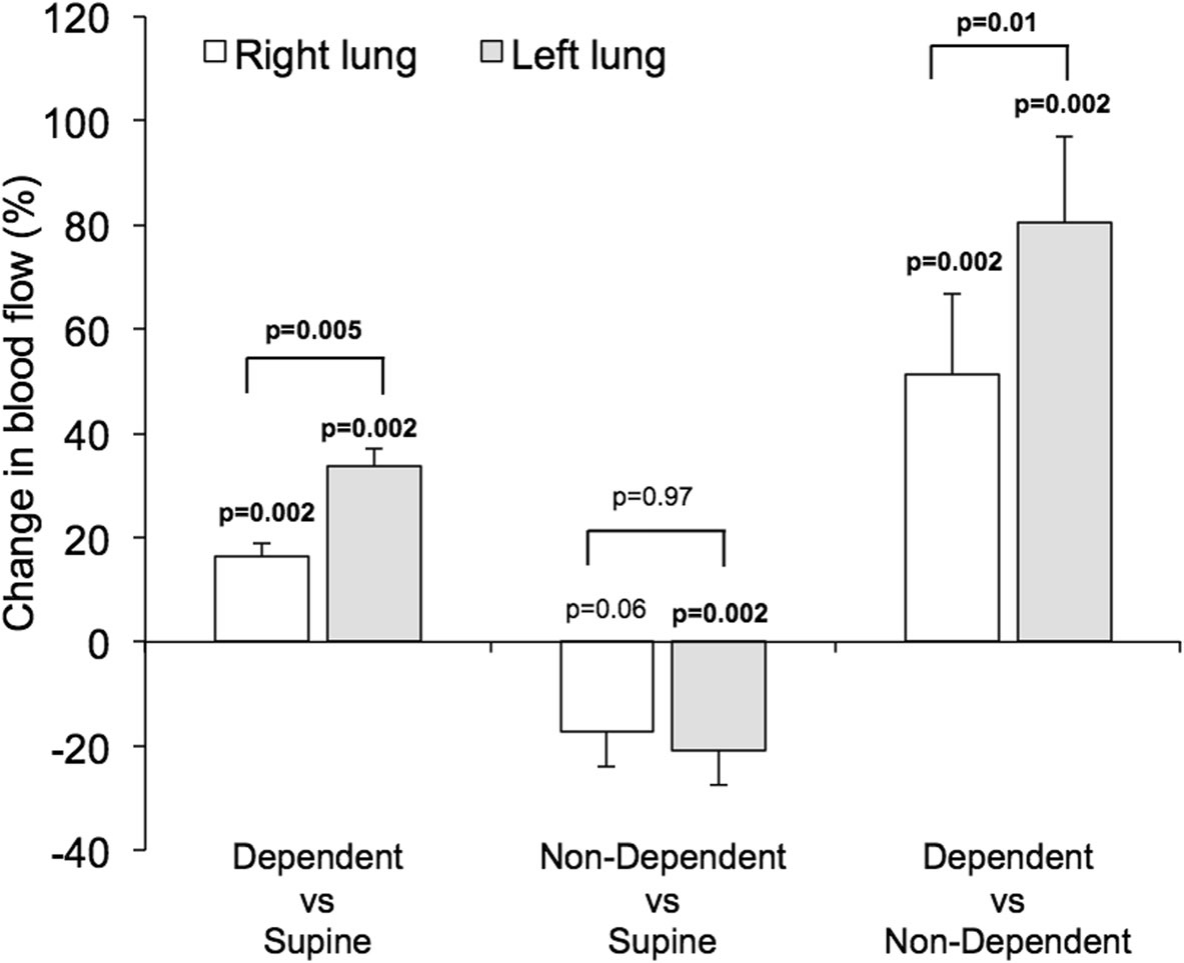
Bars show the percent change in blood flow between body positions for the right lung (white bars) and the left lung (gray bars). Blood flow is greatly increased to the dependent lung compared to non-dependent lung. The P-values placed directly above bars reflect the hypothesis that a given bar has a non-zero value, i.e. that there is a difference in blood flow to a given lung induced by change of body position. The P-values placed above brackets reflect the comparison between the right versus in the left lung

The impact of body position on right/left pulmonary blood flow distribution is summarized in Fig. 3, and absolute flow values per lung are presented in Fig. 4. In the supine and prone positions, there was a slightly higher blood flow to the right lung (supine: *p* = 0.002, prone: *p* = 0.001), with a 52/48 ± 1% right/left balance in the supine position. In both lateral positions, the dependent lung received more blood (right lateral position: p = 0.002, left: p = 0.001). In the right lateral position, the right/left distribution of blood flow changed to 63/37 ± 2% (*p* < 0.002 vs supine). Correspondingly, the left lateral position altered the right/left distribution of blood flow to 41/59 ± 3% (*p* < 0.01 vs supine). The percent change in blood flow per lung and body position is presented in Fig. 5. Changing from the non-dependent or the supine position to the dependent position led to a percent increase in blood flow that was larger to the left lung (*p* ≥ 0.01 for both changes in body position).

### Impact of body position on pulmonary venous flow distribution

**Fig. 6:**
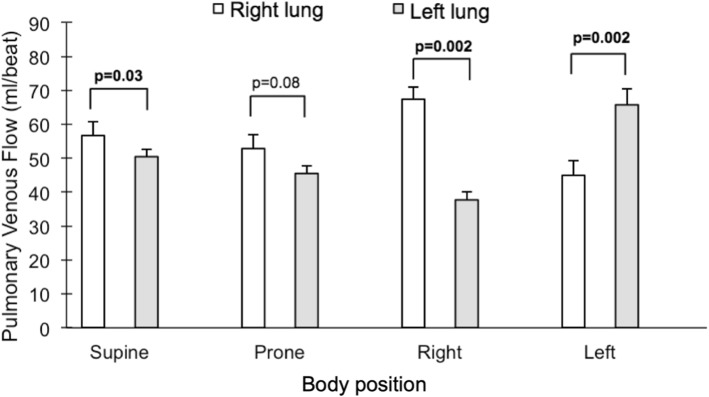
Comparison between the pulmonary venous flow volumes in each body position for the right lung (white bars) and the left lung (gray bars). Error bars represent standard error of the mean (SEM)

**Fig. 7:**
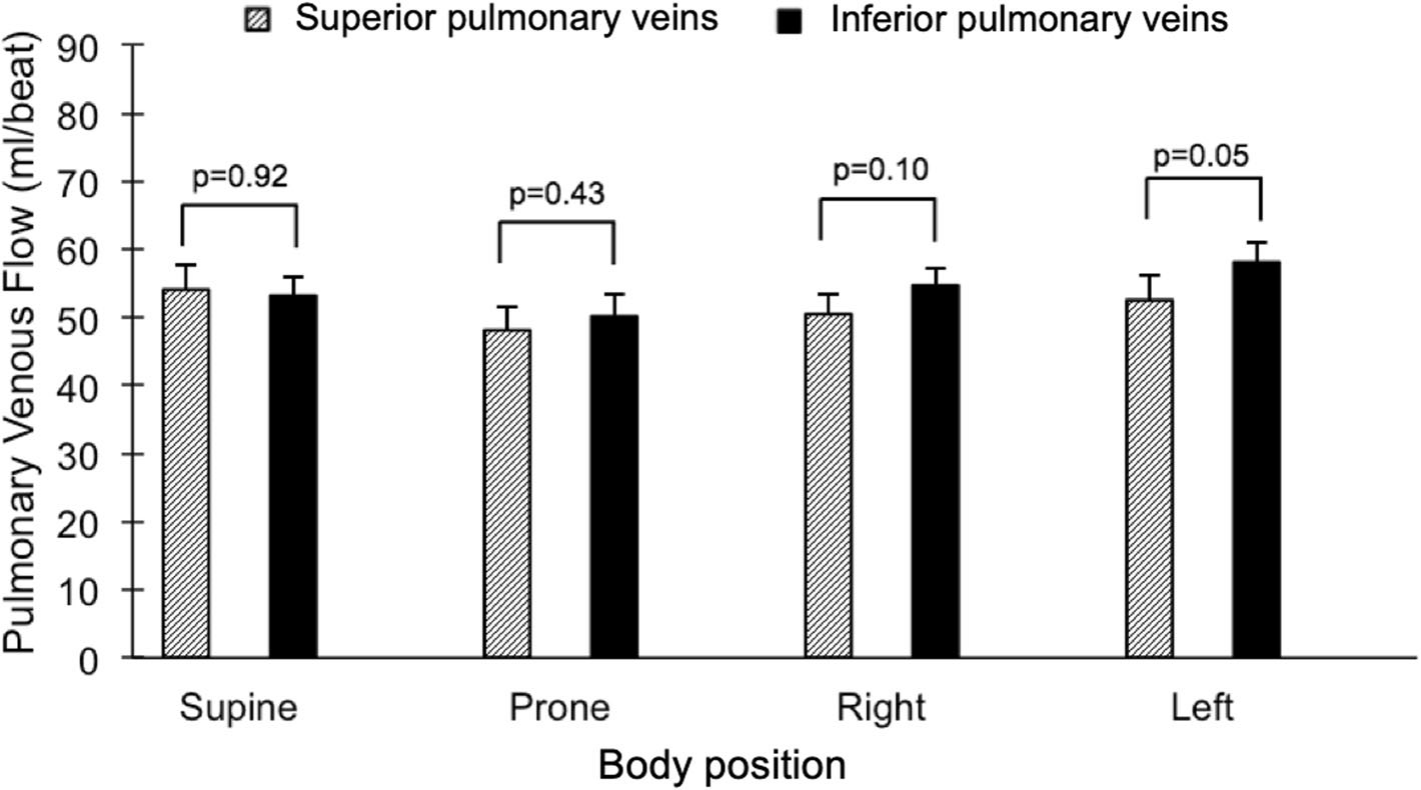
Comparison between the superior (slanted lines) and inferior (black) pulmonary venous flow volumes in each body position. Error bars represent standard error of the mean (SEM)

The summed flow in both right and left pulmonary veins, respectively, in each body position showed similar findings to arterial flow with regards to right/left balance, with the dependent lung flow contributing 64 ± 2% and 60 ± 3%, respectively, of venous flow in the lateral positions (*p* = 0.002 for both sides, Fig. 6). In contrast to the arterial flow, there was a difference between right and left venous flow in the supine, but not the prone, position (*p* = 0.03 and *p* = 0.08, respectively). Right/left venous flow distribution in the supine position was 53/47 ± 1%, *p* = 0.03. We further compared the summed blood flow of the left and right superior pulmonary veins, draining the upper and right middle lobes of the lungs, to the summed blood flow of the left and right inferior pulmonary veins draining the lower lobes of the lungs. There was no detectable difference between the superior and inferior pulmonary venous flow, *p* ≥ 0.05 for all four body positions (Fig. 7). Notably, the drainage from the inferior pulmonary veins was slightly larger than from the superior veins in the left lateral position, *p* = 0.05. While this difference bordered on achieving statistical significance, the magnitude of difference was small (6 ml or 11%).

The mean ± SD difference between main pulmonary arterial flow and summed pulmonary venous net flow ranged between 0 ± 7 ml (0 ± 7%) and 4 ± 4 ml (4.0 ± 3.8%) across the four body positions.

### Impact of body position on cross-sectional vessel areas

**Fig. 8:**
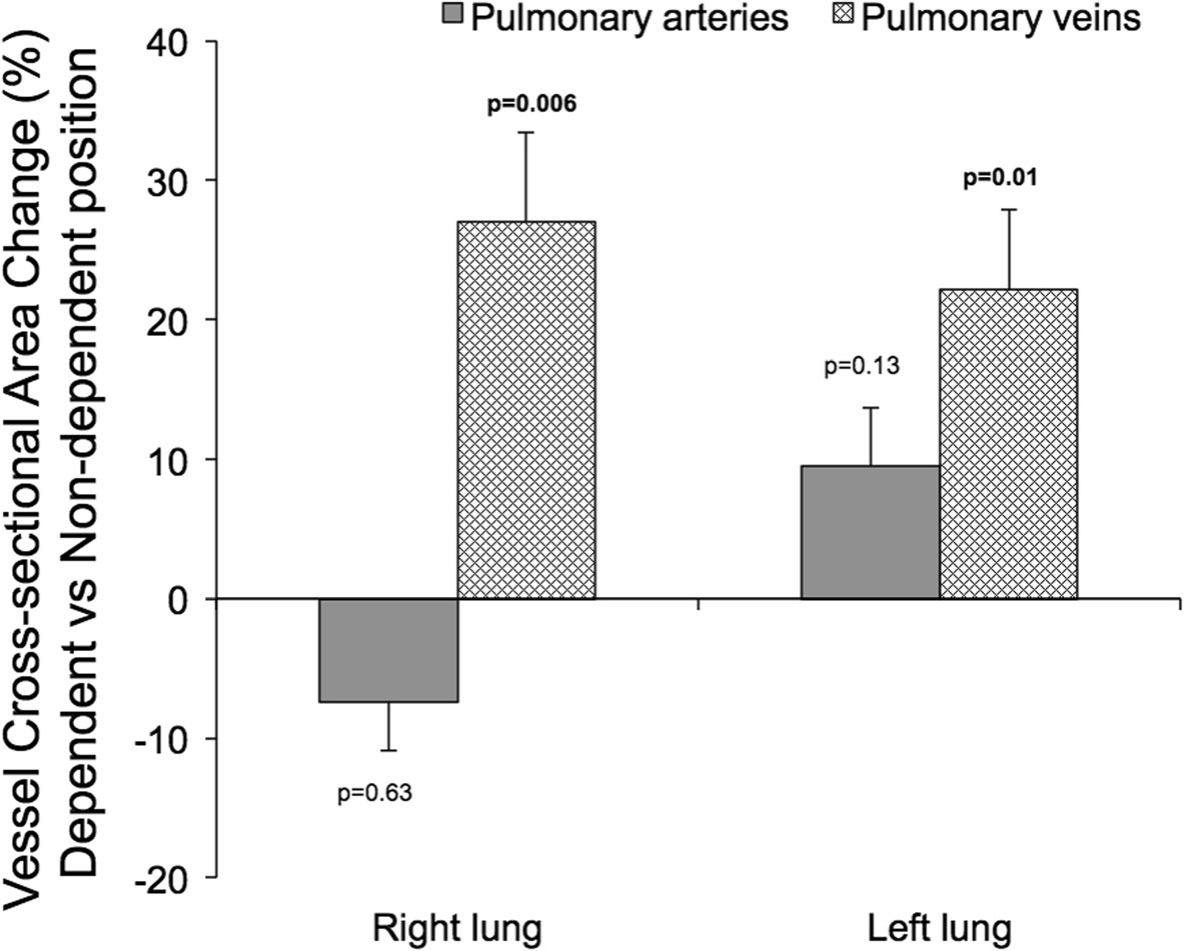
The percent change in average cross-sectional vessel area during the cardiac cycle in the dependent compared to the non-dependent position. The dark grey bars represent the percent change in the pulmonary arterial areas for the right and left lung, respectively. The chequered bars represent the percent change in the sum of the areas for the superior and inferior veins in the right and left lung, respectively. The venous cross-sectional area increased in the dependent position compared to when the same lung was in the non-dependent position. The arterial area remained unchanged with body position. The *P*-values reflect the hypothesis that bars have a non-zero value, i.e. that vessel cross sectional area is affected when a lung goes from being dependent in the lateral position to being non-dependent in the contralateral position

**Fig. 9:**
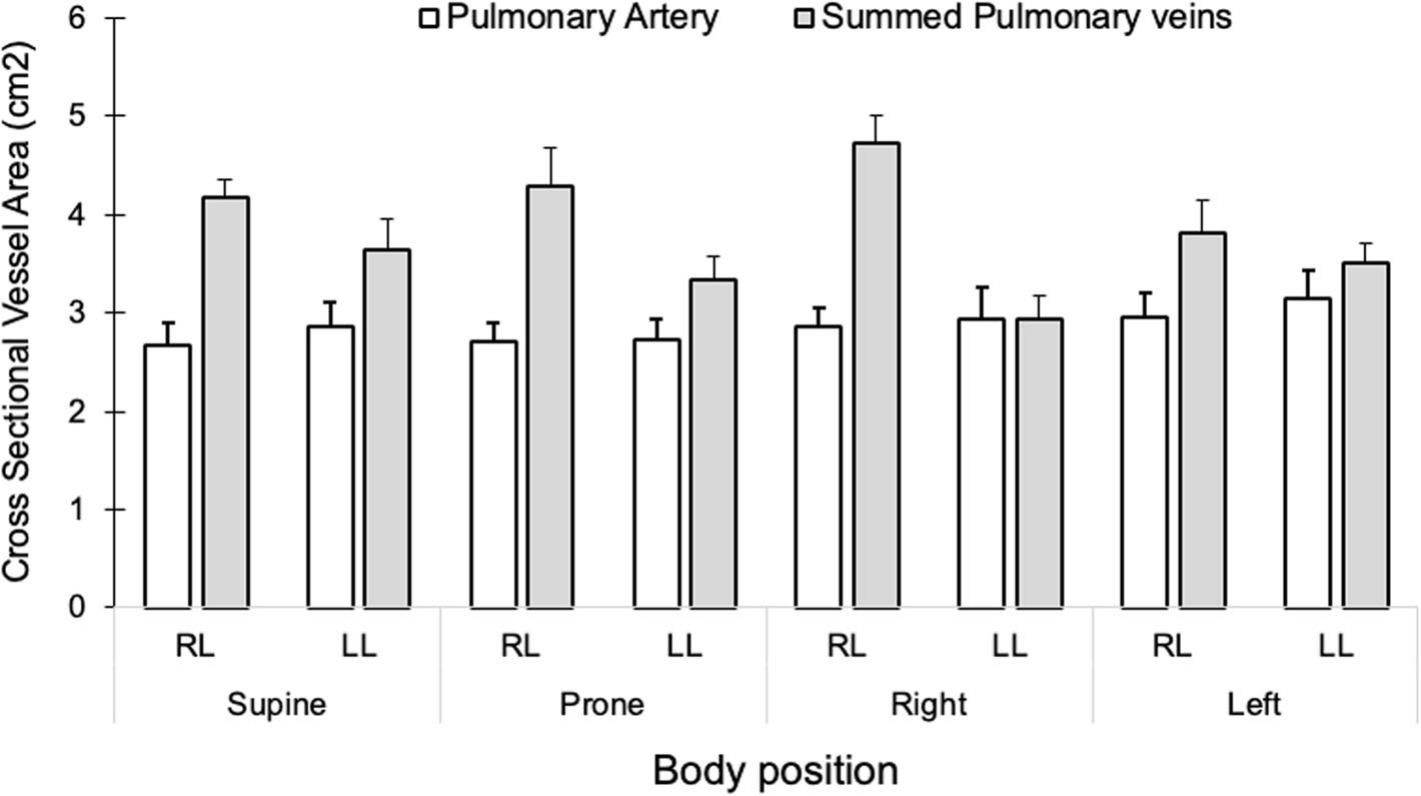
The mean cross-sectional vessel areas, averaged over the heart cycle, are shown for the pulmonary artery (white bars) and the combined superior and inferior pulmonary veins (gray bars) for the right and left lungs in each investigated body position. Error bars represent the standard error of the mean. Abbreviations: LL – left lung. RL – Right lung

The relative changes in arterial and venous vessel cross-sectional areas in the non-dependent compared to the dependent position are shown in Fig. 8. Vascular cross-sectional area averaged over the cardiac cycle did not differ for the pulmonary arteries between the non-dependent and the dependent position for either lung (*p* > 0.13 for both). However, pulmonary venous cross-sectional area was greater in both lungs when in the dependent compared to the non-dependent position (*p* ≤ 0.01 for both). Absolute values for arterial and venous cross-sectional areas are shown in Fig. 9.

### Impact of body position on absolute PBVV

**Fig. 10:**
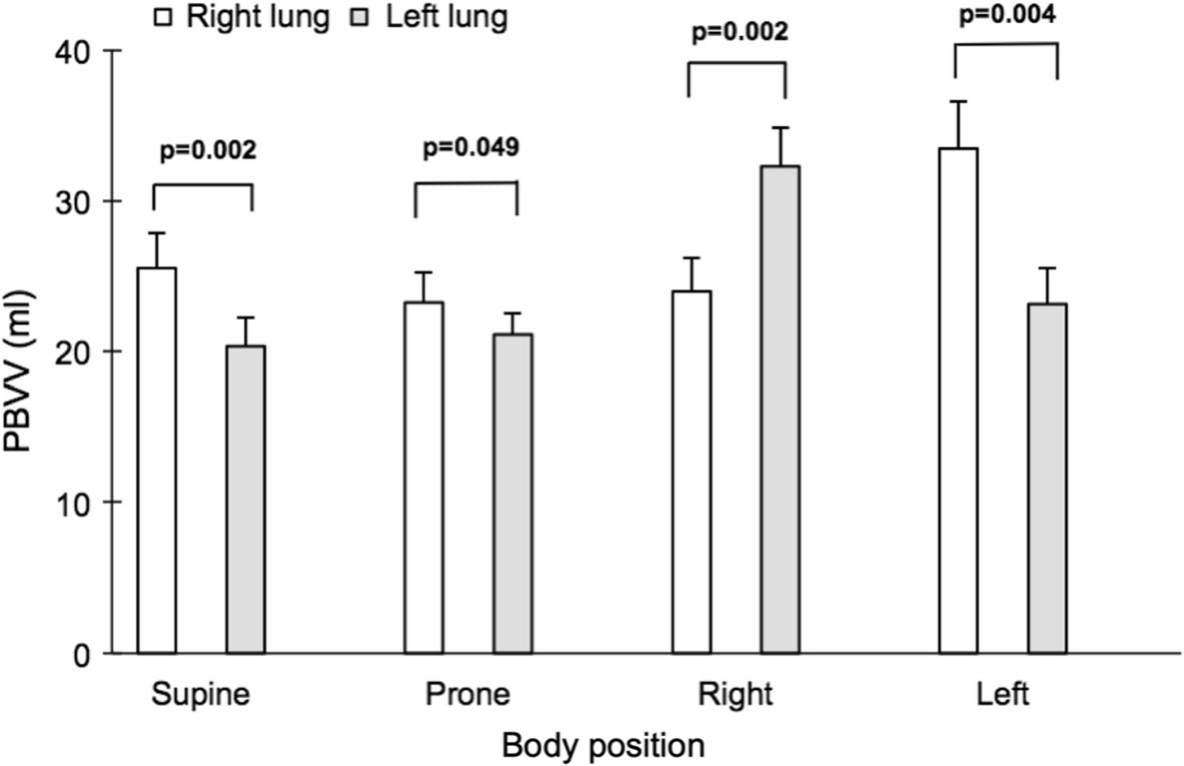
Comparison between PBVV in each body position for the right lung (white bars) and the left lung (gray bars). Error bars represent standard error of the mean (SEM)

Bilateral PBVV was 46 ± 4 ml or 44 ± 2% of the stroke volume in the supine position. PBVV in the right lung was higher than PBVV in the left lung in the supine position (26 ± 2 vs 20 ± 2 ml, *p* = 0.002), while right and left PBVV showed less prominent differences in the prone position (23 ± 2 vs 21 ± 1 ml, *p* = 0.049), Fig. 10. In both lateral positions, PBVV was markedly higher in the non-dependent lung. This was particularly pronounced in the right lateral decubitus position, where PBVV in the non-dependent left lung was higher than PBVV in the dependent right lung (left 32 ± 3 ml vs right 24 ± 2 ml, *p* = 0.002). The corresponding pattern was also found in the left lateral position, with greater PBVV in the non-dependent lung (*p* = 0.004).

### Impact of body position on relative PBVV

**Fig. 11:**
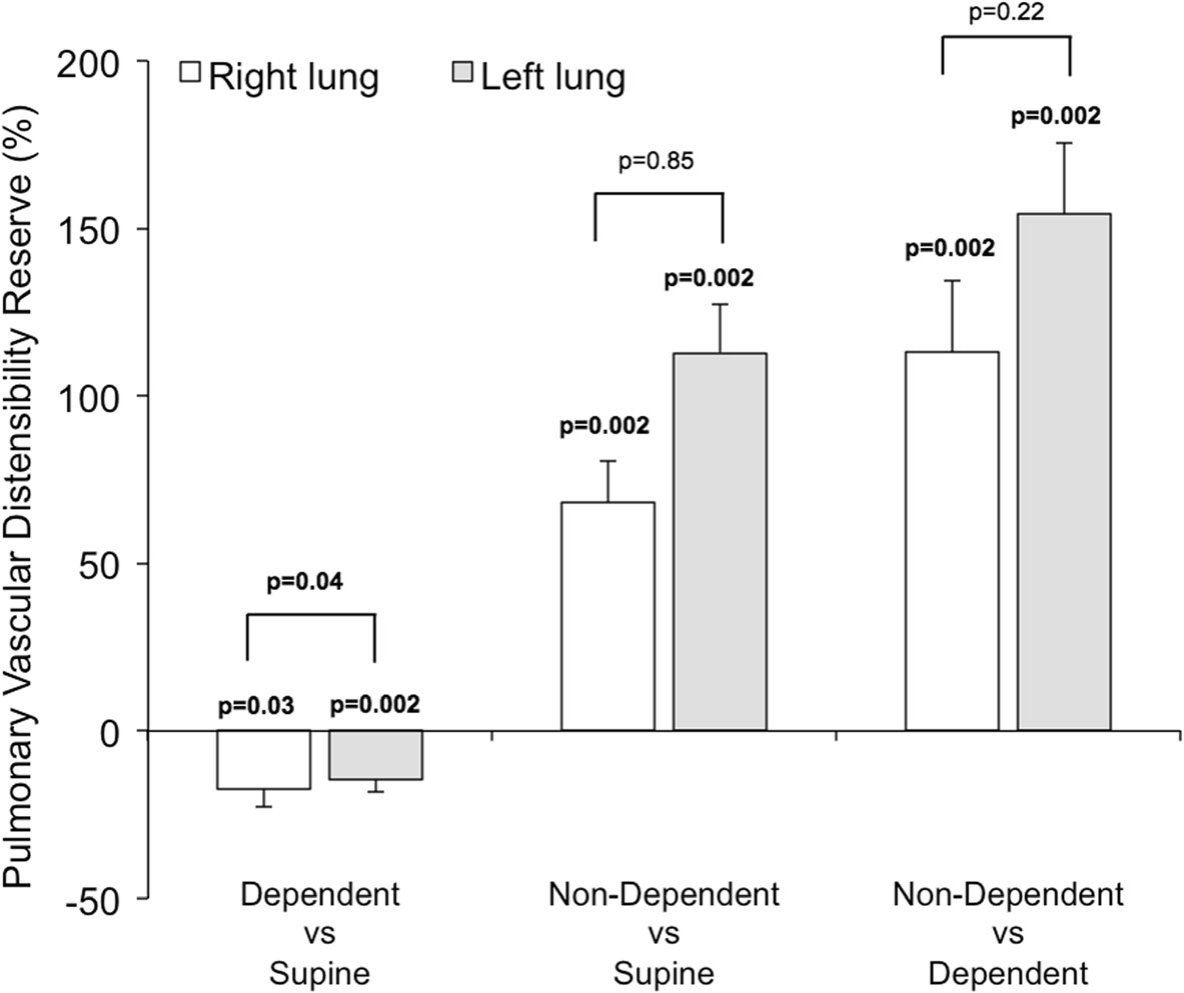
The pulmonary vascular distensibility reserve (PVDR), defined as the percent change in relative pulmonary blood volume variation (PBVV) in the right lung (white bars) and the left lung (gray bars). The PVDR is greatly increased in the non-dependent position on both sides, owing to an increase in absolute PBVV and a decrease in ipsilateral arterial flow. The P-values placed directly above bars reflect the hypothesis that a given bar has a non-zero value, i.e. that the PVDR is non-zero for a given pair of body positions. The P-values placed above brackets reflect the difference between the right and left lung

**Fig. 12:**
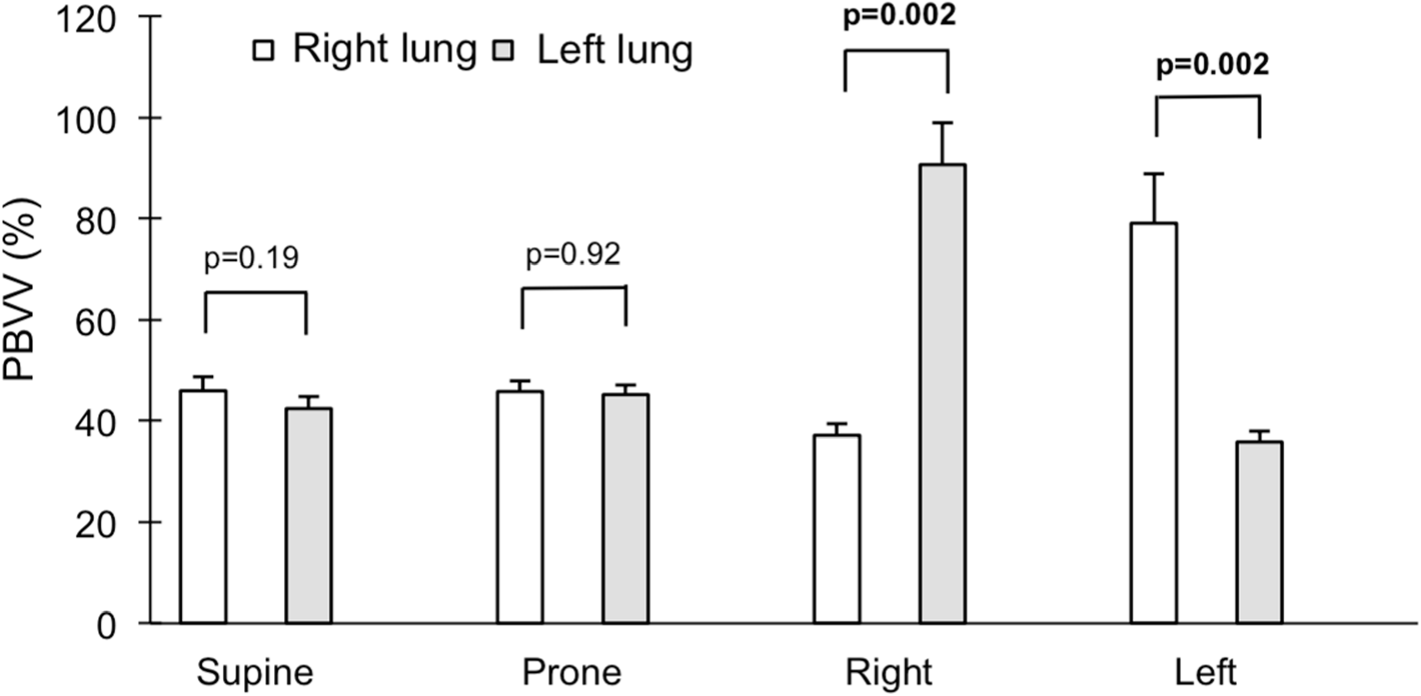
Comparison between PBVV indexed to ipsilateral blood flow volume per beat in each body position for the right lung (white bars) and the left lung (gray bars). Error bars represent standard error of the mean (SEM)

In both lateral positions, the difference in dependent vs non-dependent relative PBVV was large due to the combination of greater absolute PBVV and lesser ipsilateral blood flow to the non-dependent lung. The difference was most pronounced in the right lateral decubitus position, where relative PBVV to the non-dependent left lung was 54 percentage points higher than to the dependent right lung (right: 37 ± 2% vs left: 91 ± 8%, p = 0.002). The percent change in relative PBVV per lung and body position is visualized in Fig. 11, and the magnitude of relative PBVV is summarized in Fig. 12. On average, increase in relative PBVV, termed pulmonary vascular distensibility reserve (PVDR), was 113 ± 15% in the left lung when in the non-dependent position versus in the supine position, *p* = 0.002. In the right lung, the corresponding PVDR was 68 ± 12%, p = 0.002.

### Interobserver variability

**Fig. 13:**
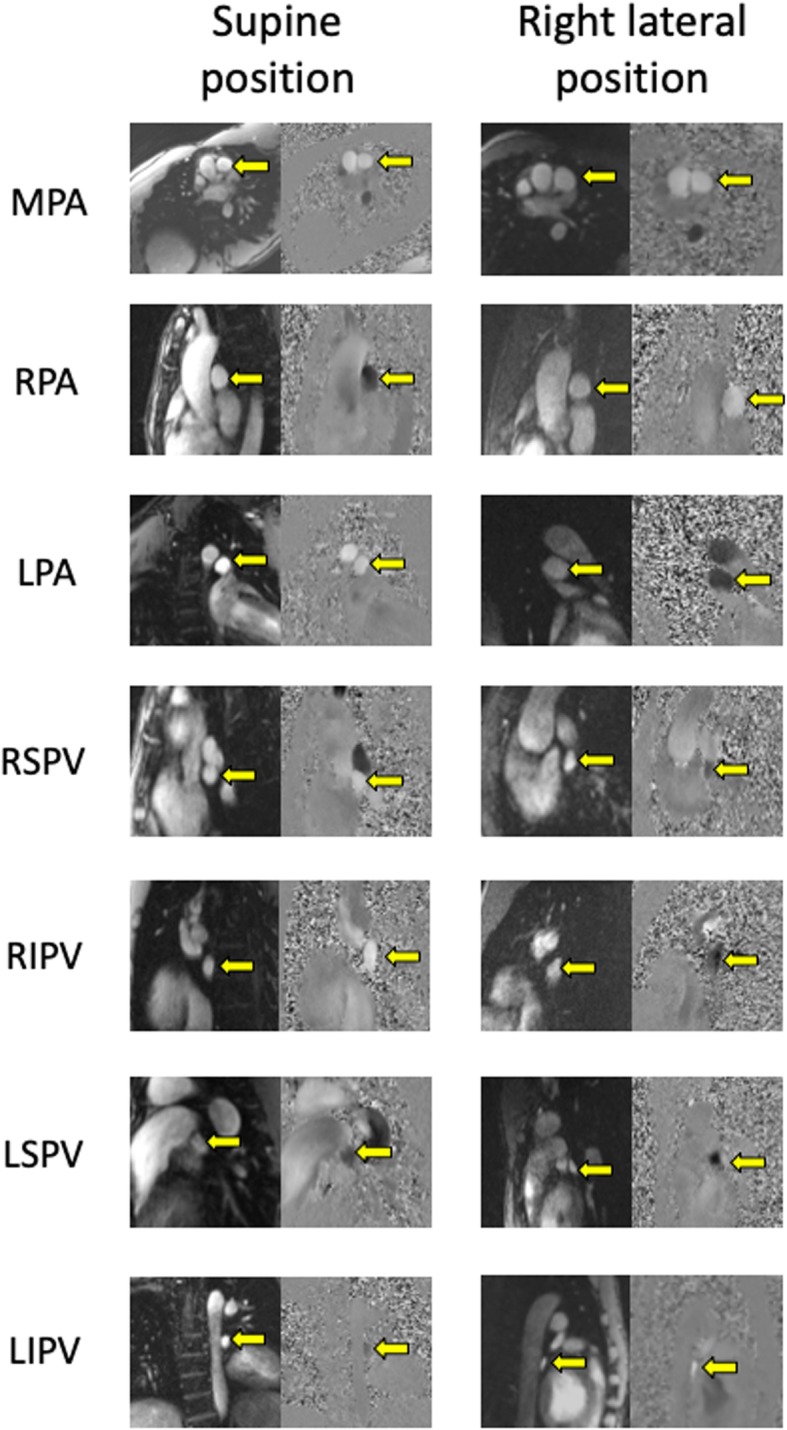
Cross-sectional screenshots of the pulmonary vessels from magnitude and phase contrast images obtained from a representative subject using a commercially available free-breath 2D flow sequence with three averages. Images obtained in the supine position are shown in the left column. Images obtained in the right lateral position are shown in the right column. Targeted vessels are marked with a yellow arrow. Abbreviations: LIPV = Left Inferior Pulmonary Vein. LPA = Left Pulmonary Artery. LSPV = Left Superior Pulmonary Vein. MPA = Main Pulmonary Artery. RIPV = Right Inferior Pulmonary Vein. RPA = Right Pulmonary Artery. RSVP = Right Superior Pulmonary Vein

For PBVV, the mean percent difference from the average value of the two observers was 4.8 ± 4.3% for the left lung in the supine position, and 2.0 ± 1.5% for the left lung in the right lateral position. The ICC for PBVV was 0.89 for the left lung in the supine position, and 0.98 for the left lung in the right lateral position. The Dahlberg error was 1.9 ml (relative Dahlberg error: 8.9%) for the left lung in the supine position, and 1.2 ml (relative Dahlberg error: 3.5%) for the left lung in the right lateral position. The corresponding numbers for the relative PBVV of the left lung were: 6.8 ± 7.1% (right lateral position: 5.3 ± 4.5%), Dahlberg error: 6.5% of SV (right lateral position: 8.5% of SV), relative Dahlberg error: 13.5% (right lateral position: 9.6%) and an ICC of 0.44 (right lateral position: 0.89). For PVDR for the left lung changing from the supine into the right lateral position, the mean percent difference was 21.6 ± 23.1%, Dahlberg error: 35.4%, relative Dahlberg error: 43.6% and ICC: 0.62. Magnitude and phase-contrast images from all imaged vessels from one representative case are shown in Fig. 13.

## Discussion

**Fig. 14:**
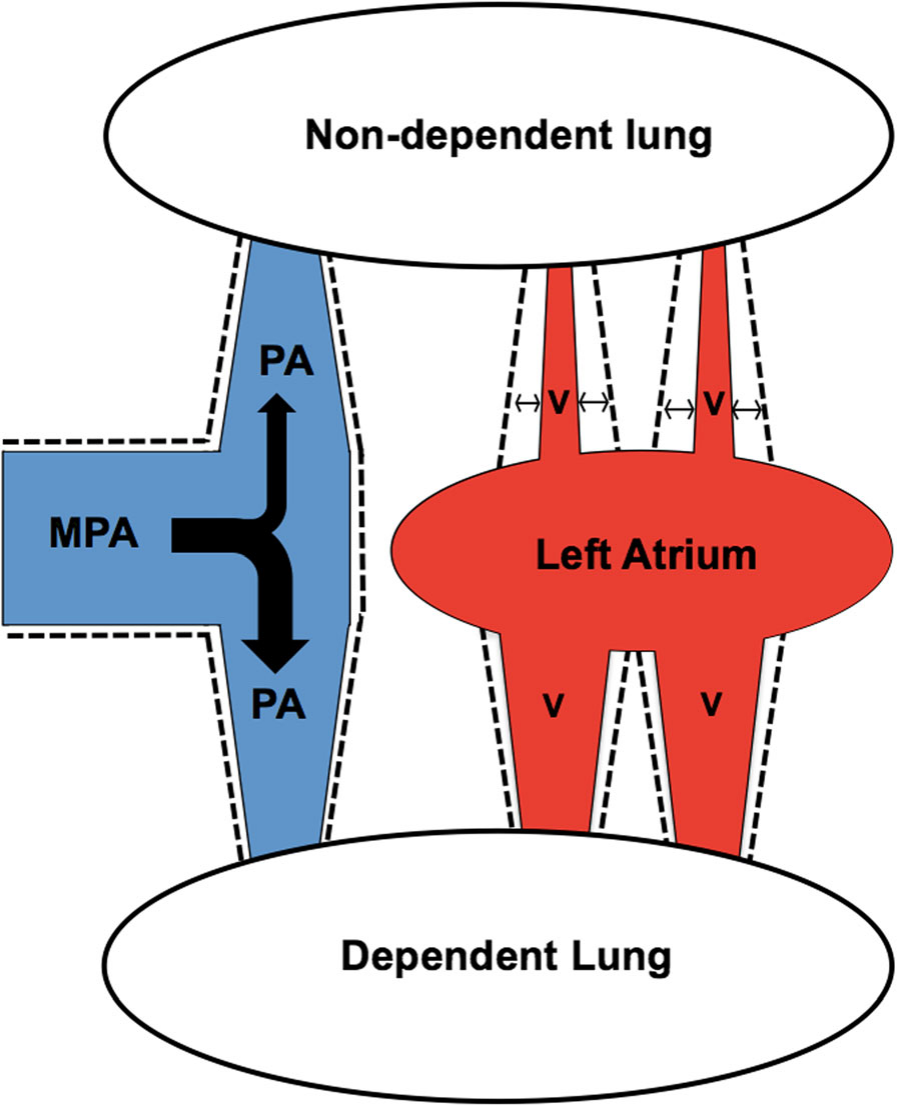
A schematic illustration of the effects of the lateral decubitus position upon pulmonary circulatory hemodynamics in healthy subjects. The dependent lung receives the majority of blood flow at the expense of a decrease in flow to the non-dependent lung, as illustrated by the thickness of the black bifurcating arrow in the main pulmonary artery (MPA) to the respective pulmonary arteries (PA). The pulmonary veins (V) in the dependent lung are more distended compared to those in the non-dependent lung, as illustrated by the greater diameter of the red veins coming from the dependent lung compared to those coming from the non-dependent lung. By comparison, there is no difference in vessel distension on the arterial side, as illustrated by the equal diameters of the two pulmonary arteries (PA). The vascular distensibility, as illustrated by the dotted contours and the horizontal arrows, is much higher in the non-dependent lung in particular in the non-dependent veins, and this is related to the lesser pulmonary venous distension in the non-dependent lung

The major finding of this study is that the lateral position increases pulmonary blood flow to the dependent lung by approximately 25% compared to the supine position, and profoundly increases pulmonary vascular distensibility of the non-dependent lung. This is likely mediated by a lesser pulmonary venous distension in the non-dependent lung compared to the dependent lung, as indicated by changes in cross-sectional venous areas. These findings are summarized and illustrated schematically in Fig. 14.

### Positional effects upon the pulmonary circulation

We found a right/left distribution of pulmonary blood flow of 54/46% in the supine position, shifting to 63/37% in the right lateral position and 41/59% in the left lateral position. We further found a larger increase in blood flow to the left lung in the dependent position, possibly due to anatomical right/left differences in vessel tree architecture and/or lung size.

Anesthesiology textbooks [6, 7] cite known measures of right/left pulmonary blood flow distribution according to body position in humans. These textbooks state that the right/left distribution of pulmonary blood flow is 55/45% in upright/supine, 65/35% in right lateral position and 45/55% in the left lateral position [6, 7], with references to studies in dogs, primates or humans [8, 10, 11, 20, 21]. These studies used varying respiratory conditions with regards to subjects breathing spontaneously, or being anesthetized and intubated with varying positive airway pressures. Importantly, these studies utilized varying methods for measuring blood flow including the use of radiotracers or extrapolations from oxygen consumption (VO_2_) measurements in small sample sizes, and show somewhat diverging results. In concordance with the results of the present study, one study found a 43/57% right/left distribution of pulmonary perfusion in the left lateral position using intravenous injection of Xe^133^ and measurement of the exhaled concentration of Xe^133^ in a double lumen endotracheal tube in patients without cardiopulmonary disease [9]. In contrast, a different study found a close to balanced distribution between the dependent and non-dependent lungs in both lateral positions, with most subjects having a majority of blood flow to the dependent lung with a few extreme outliers [22]. Those authors speculated that this may be due to Xe^133^ flowing through shunts in atelectases in the dependent lung not being exhaled. Other studies have been performed to evaluate the effects of gravity in the lateral position on the pulmonary circulation using positron emission tomography (PET) or pulmonary scintigraphy [23, 24]. These studies indicate that blood flow tends to favor dependent regions of the lungs, but showed that gravity has a lesser impact particularly in the prone position. Taken together, prior results have been inconclusive with varying results in limited sample sizes and potentially imprecise methods. The current study is the first study to use robust methods to provide definitive data on the magnitude of the effects of the lateral position on pulmonary blood flow distribution in spontaneously breathing awake humans.

### Positional impact on superior/inferior venous flow

Our results show that the supine and prone positions did not affect the balance between flow in the ventral upper lung lobes drained by the superior pulmonary veins, and the more dorsal lower lung lobes drained by the inferior pulmonary veins. This is in agreement with the above-mentioned nuclear imaging studies showing no meaningful perfusion gradient between dorsal and ventral lobes in the prone position [23–25]. This suggests that the improved gas exchange observed in the prone position compared to supine [1] is unrelated to gravity-induced redistribution of blood flow from the lobes drained by the superior and inferior pulmonary veins, respectively. Although the difference in superior and inferior venous flow volume approached reaching statistical significance in the left lateral position (*p* = 0.05), we see no reasonable physiological explanation behind this finding. Furthermore, the magnitude of the superior/inferior venous flow difference did not differ in the right lateral position, indicating that the observed difference in the left lateral position may be due to statistical noise.

### Mechanism of positional effect on pulmonary blood flow distribution

The three-zone model initially developed by West, et al. [26], describes the impact of gravity on the pulmonary circulation. One mechanism governing this model is the gravity-induced differences in hydrostatic pressures which affect vessel distension and therefore regional vascular resistance and blood flow. Further studies have demonstrated large heterogeneity in blood flow within isogravitational planes [1]. This has led to the conclusion that vessel anatomy is the major determinant of regional pulmonary blood flow, and that gravity is an important but secondary determinant [27]. Our results strongly suggest that gravity plays a role in determining the distribution of blood flow between the dependent and non-dependent lungs in the lateral positions. Importantly, our study is the first to provide data to support the notion that there is a redistribution of blood volume in the pulmonary venous circulation that coincides with the changes in blood flow.

### Reproducibility of PBVV

We found the combined PBVV in both lungs to be roughly 50 ml and 50% of stroke volume (46 ± 4 ml, 44 ± 2%), and this agreed excellently with a previous study of healthy subjects in the supine position (48 ± 5 ml, 46 ± 6%) [15]. This suggests that the method to determine PBVV by CMR is reproducible across different healthy populations, sites, CMR scanner vendors, and operators. Our findings regarding inter-observer variability were found to be consistent with the findings of a previous study that reported a 1 ± 6% inter-observer variability for PBVV [16].

There are several sources for measurement error in using 2D flow measurements which are important to minimize in order to avoid large compounded measurement errors from several vessels. We attempted to minimize error through carefully placed imaging planes perpendicular to the vessel course, eddy current compensation with careful static tissue masking and manual review of automated vessel delineation in all time frames. This resulted in an average difference between pulmonary arterial and summed venous flow in our cohort that we believe is acceptable for all body positions.

### Physiology and potential diagnostic utility of PBVV

Our results demonstrate that PBVV varies with body position to a greater extent than does ipsilateral blood flow, and that this appears to be related to gravity-induced variations in distension of the pulmonary veins rather than the pulmonary arteries. The determinants of PBVV and their relative importance have yet to be explored, since PBVV is a relatively recently described measure. We hypothesize that important determinants of PBVV include the combined arterial and venous vessel wall compliance, circulating blood volume and its distribution measured as vessel distension, and blood flow volume per heartbeat. The non-dependent lung should experience decreased hydrostatic pressure due to gravity, and receive less blood, translating into decreased venous distension. This is supported by our finding of decreased venous cross-sectional area in the non-dependent position compared to the same lung in the dependent position. Due to the relative lack of smooth muscle in the walls of the pulmonary veins, the vessel wall compliance of pulmonary veins can be expected to be high, and thus pulmonary venous blood volume should be sensitive to pressure changes induced by the effects of gravity on blood volume and blood flow distribution related to body position. Taken together, our findings support the notion that PBVV is a marker inversely related to pulmonary venous distension. This is consistent the previous finding that LV dysfunction in swine reduces PBVV [2], possibly through increased left atrial pressure leading to congestion and distension of the pulmonary veins. Notably, in that study of experimental infarction [2], pulmonary wedge pressure was increased after infarction, and this is consistent with increased left atrial pressure contributing to a decrease in PBVV. This implies that PBVV could potentially be used to non-invasively gauge left atrial pressure. Specifically, we speculate that increased left atrial pressure may lead to a smaller change in relative PBVV with body position. Thus, left atrial pressure would be inversely related to PVDR, in concordance with the expectation that an increase in left atrial pressure would increase pulmonary venous distension and decrease the distensibility reserve. Notably, this hypothesis remains to be evaluated and tested prospectively.

The potential clinical role of PC-CMR in characterizing pulmonary hypertension is tantalizing in light of the findings that 4D flow PC-CMR can accurately, precisely, and non-invasively measure mean pulmonary arterial pressure [14]. Taken together, PC-CMR could potentially be used to both identify pulmonary hypertension as such, and also possibly to characterize it as pre- or post-capillary in nature, based on estimation of left atrial pressure by measuring PVDR.

It remains to be determined whether a 4D flow approach could be useful in measuring PBVV and PVDR in a way that is less dependent on the operator of the CMR scanner, and equally as accurate and precise as for 2D PC imaging.

### Limitations

An important limitation of this study was the limited sample size which was adequate to glean results with regards to physiology, but insufficient to measure normal references ranges for PBVV and PVDR in healthy subjects. Another limitation was that although our inclusion criteria permitted a variety of ages, the subjects most accessible to us were young and healthy which is reflected in our cohort. Thus, the resulting homogenous cohort did not permit studying the effects of normal aging and disease on PBVV and PVDR, which remain to be explored. The presence of atelectasis may potentially influence both PBVV and PVDR. In future studies exploring the utility of PBVV and PVDR in subjects with pulmonary disease, it may be necessary to develop strategies to account for atelectasis*.* Furthermore, an important limitation is that conditions causing irregular heart rate, such as atrial fibrillation or excessive premature contractions, preclude the accurate measurement of PBVV and PVDR due to their detrimental effect on the reliability of CMR flow measurements.

In addition, the test-retest repeatability of PBVV, relative PBVV and PVDR remain to be studied in order to quantify the differences in these measures that can be accurately measured. An additional limitation is that observed venous vessel areas can be influenced by the placement of the image plane closer to or further away from the left atrium, due to the pronounced tapering of pulmonary veins.

In case of pulmonary venous abnormalities and individual variations, these would have to be taken into account when measuring PBVV. Additional pulmonary veins may require separate imaging planes, while certain shunt variations may make the measurement of PBVV unreliable for some patients.

### Clinical implications and future directions

Our results provide quantitative data on blood flow distribution that help explain the drop in partial oxygen pressure seen in patients with acute respiratory distress syndrome (ARDS) in the lateral position, with the most affected lung in the dependent position [26, 28]. Lateral position PBVV, and PVDR, are novel measures which may theoretically correlate with left atrial pressure through venous distension. Measurement of left atrial pressure, LV end-diastolic pressure, or pulmonary wedge pressure are invasive, associated with risk, and difficult to motivate measuring in healthy volunteers. Therefore, the use of PVDR to non-invasively distinguish between pre- and postcapillary pulmonary hypertension by estimating left atrial pressure should be investigated in patients undergoing clinical invasive pressure measurements, and such studies are underway.

## Conclusions

This study is the first to provide definitive data confirming widespread notions of right/left pulmonary blood flow distribution in the lateral position. Furthermore, we corroborate previous findings of pulmonary vascular distensibility, measured as PBVV in healthy subjects, suggesting robust reproducibility of the method. We conclude that there is an increase in PBVV in the non-dependent lung compared to when the same lung is in the supine position. The positional increase in PBVV in the non-dependent lung is more pronounced when the blood flow volume to the same lung is taken into account. We introduce the novel measure PVDR to describe this positional change in relative PBVV. Also, positional changes in cross-sectional vessel areas suggest that PVDR is due to gravity-induced differences in venous, but not arterial, distension between the dependent and non-dependent lung. Thus, PVDR may be related to left atrial pressure. Future studies are warranted to evaluate the potential clinical role of these novel measures, primarily in the context of characterizing pulmonary hypertension.

## Data Availability

The datasets used and/or analyzed during the current study are available from the corresponding author on reasonable request.

## Abbreviations

ARDS: Acute respiratory distress syndrome
BMI: Body mass index
BSA: Body surface area
bSSFP: Balanced steady-state free precession
CMR: Cardiovascular magnetic resonance
ECG: Electrocardiogram
LV: Left ventricle/left ventricular
LVEDV: Left ventricular end-diastolic volume
LVEDVI: Left ventricular end-diastolic volume index
LVEF: Left ventricular ejection fraction
LVM: Left ventricular mass
LVMI: Left ventricular mass index
PBVV: Pulmonary blood volume variation
PC: Phase contrast
PET: Positron emission tomography
PVDR: Pulmonary vascular distensibility reserve
VO_2_: Oxygen consumption
